# Size-Class Effect Contributes to Tree Species Assembly through Influencing Dispersal in Tropical Forests

**DOI:** 10.1371/journal.pone.0108450

**Published:** 2014-09-24

**Authors:** Yue-Hua Hu, Roger L. Kitching, Guo-Yu Lan, Jiao-Lin Zhang, Li-Qing Sha, Min Cao

**Affiliations:** 1 Key Laboratory of Tropical Forest Ecology, Xishuangbanna Tropical Botanical Garden, Chinese Academy of Sciences, Mengla, Yunnan, China; 2 Environmental Futures Research Institute, Griffith School of the Environment, Griffith University, Nathan, QLD, Australia; 3 Rubber Research Institute, the Chinese Academy of Tropical Agricultural Sciences, Danzhou, Hainan, China; The Ohio State University, United States of America

## Abstract

We have investigated the processes of community assembly using size classes of trees. Specifically our work examined (1) whether point process models incorporating an effect of size-class produce more realistic summary outcomes than do models without this effect; (2) which of three selected models incorporating, respectively environmental effects, dispersal and the joint-effect of both of these, is most useful in explaining species-area relationships (SARs) and point dispersion patterns. For this evaluation we used tree species data from the 50-ha forest dynamics plot in Barro Colorado Island, Panama and the comparable 20 ha plot at Bubeng, Southwest China. Our results demonstrated that incorporating an size-class effect dramatically improved the SAR estimation at both the plots when the dispersal only model was used. The joint effect model produced similar improvement but only for the 50-ha plot in Panama. The point patterns results were not improved by incorporation of size-class effects using any of the three models. Our results indicate that dispersal is likely to be a key process determining both SARs and point patterns. The environment-only model and joint-effects model were effective at the species level and the community level, respectively. We conclude that it is critical to use multiple summary characteristics when modelling spatial patterns at the species and community levels if a comprehensive understanding of the ecological processes that shape species’ distributions is sought; without this results may have inherent biases. By influencing dispersal, the effect of size-class contributes to species assembly and enhances our understanding of species coexistence.

## Introduction

The way in which size-class influences the assembly of tree species within communities is important to species coexistence. For tree species with long lifespans, many attributes may vary across size-classes including spatial patterns [1], [2], [3], habitat associations [4], [5], [6], physiological requirements and selective pressures [7], [8]. As a result, mechanisms of species assembly may change as trees age [9], [10]. Several theories have been proposed to explain species coexistence [11], [12], [13], [14], but no unified theory has emerged at this time [15]. Many recent empirical studies have identified deterministic ‘niche’ and stochastic ‘neutral’ assembly as the two principal mechanisms shaping tree species coexistence either separately or in conjunction [1], [16], [17], [18]. An exploration of the size-class effect on niche and neutral processes should provide an robust evaluation of the impact of size-class on species assembly. Although a few studies have examined the relative importance of niche and neutral processes at specific size-classes [5], [6], [10], [19], [20], there has been no study to date that models, directly, the impact of size-class on species assembly.

The long-term monitoring of the dynamics of tree species is a direct way of examining the role of size-class in species assembly yet the extended life span of trees makes this challenging. Examining the standing size structure and the size-specific spatial patterns of tree species is a more practical alternative of achieving, at least in part, the same understanding. Models of point processes are effective for modeling the spatial distributions of tree species and may be useful, accordingly, for evaluating the roles of niche and neutral processes in species assembly [21]. Previous studies using point process models have successfully simulated all mapped trees [21], or trees within specific size-classes [19], [20] in the forest plots of interest. Hence, through modeling tree species’ distributions within specific size-classes separately, and then combining these simulations to recreate the entire community, we can effectively model size-class specific effects. Combining tree communities using different size-classes may enable us to detect complex spatial patterns at multiple clustering scales [22]. Comparative modeling of niche and neutral processes with and without size-class effect should quantify the importance of such effects in community assembly. Finally, modeling size-class effects on species assembly explicitly may lead to a novel understanding of species coexistence.

To evaluate size-class effect on species’ distributions, it is crucial to use appropriate summary characteristics to identify the differences between actual community and simulated communities using point process models. Many summary characteristics have been used in the past, including species-area relationships (SARs) [21], distance decay curves [20] and distributions of species abundances [23] for community level analysis. For analyses at the level of the individual species, statistics used have included the nearest neighbor distance function [24], Ripley’s L-function [25] and the pair correlation function [26]. Among these summary characteristics, the SAR describes the manner in which the number of species increases with increasing sampling area [27], and is related to the spherical contact distribution at the community level at relatively large scales [28]. SARs have long been examined by ecologists [29] and are a cornerstone of community ecology [30]. Wiegand *et al.* [24], however, argue that it is important to use multiple summary characteristics in order to detect the critical characteristics of complex patterns underlying species’ distributions. Because of differing dispersal abilities and different responses to environmental variables among species, Shen *et al.* [19] suggest that analysis at the species level is also necessary for the study of species’ distributions. The pair correlation function (i.e. the g-function), which represents the details of tree clustering at the level of the individual species at relatively small scales [22], [24]. is more sensitive to significant point-point interactions at specific spatial scales than many other point pattern functions [31]. It is an appropriate summary characteristic at the level of the species. Accordingly we suggest that SARs and the g-function are two ideal, mutually complementary summary characteristics for the community and species levels, respectively.

In this study, we have modeled, separately, tree distributions based on dispersal limitation, environmental limitation and a combination of both effects (based on a homogeneous Thomas process, an inhomogeneous Poisson process and an inhomogeneous Thomas process, respectively) for different DBH (diameter at breast height) size-classes. Then, by combining the simulated assemblages of different DBH classes we generate the structure of the entire community explicitly incorporating the size-class effect. Finally, we compare the SAR and g-function patterns generated for each of the two forest stands being examined using each of the three models with and without these size-class effects.

As a species may change its habitat preference and spatial point pattern at times which may corresponding to different size-class, we hypothesize that point process models with size-class effect should present much more reliable patterns of SAR and g-functions than models without size-class effects. Many previous studies have shown that the joint-effect of environment and dispersal is the principal regulator of species’ distributions. Accordingly we suggest that together these factors should play a more important role in shaping SAR and g-function patterns than the any one of the two will do, separately.

## Materials and Methods

### Ethics statement

The Management Bureau of Xishuangbanna National Nature Reserve gave us the authority to conduct tree census works in the 20-ha plot. With the authority of the principal investigator of the Barro Colorado Island (BCI) plot, we downloaded topographic data, soil data and tree data for that plot from the website of the Center for Tropical Forest Science (http://www.ctfs.si.edu/).

### Site description

Data from two seasonal tropical forests were used to investigate the mechanisms underlying the SARs and the g-functions. The first of these datasets was from the 20-ha forest dynamics plot in Bubeng village, Xishuangbanna National Nature Reserve, Southwest China (Data S1). This area is dominated by a typical monsoon climate which results in a typical dry season (November to April) and wet season (May to October). In 2007, we finished the first census of the Bubeng plot. We recorded 95,498 stems of which 468 species or morphospecies were identified, belonging to 213 genera and 70 families. The altitude of the Bubeng plot ranges from 709 to 869 m. Detailed descriptions of the climate, geology, and flora of the Bubeng plot can be found in Lan *et al.* [32] and Hu *et al.* [33]. The second dataset originated from the 50-ha forest dynamics plot on Barra Colorado Island (BCI), Panama. This plot is also located in an area with an alternation of wet and dry seasons. The plot supports lowland semideciduous moist forest with 301 species and 229,049 stems with DBH ≥1 cm. The topography of the BCI plot is flat with an altitudinal range of only 38 m. Detailed descriptions of the climate, geology, flora and fauna of the BCI plot can be found in Croat [34], Leigh *et al.* [35] and Gentry [36]. In keeping with the data from the Bubeng plot for which we used the first census data, we used the tree data from the first census of the BCI plot [37]. The tree census of both plots followed the agreed protocol for the global network of forest research plots overseen by the Center for Tropical Forest Science [38].

To quantify the effects of environmental variables on the SARs and g-functions, we used topographic and soil variables as environmental variables. Based on topographic survey data, mean elevation, convexity, aspect, and slope in each 20×20 m cell were calculated and used as topographic variables at both sites [21], [39], [40]. For the Bubeng plot, we sampled soils using a regular grid of points every 30 m, generating 252 nodes. Every second node was paired with an additional sample point at 2 m and 5 m, 2 m and 15 m or 5 m and 15 m along a random compass bearing away from its associated node. In total, 756 soil samples were taken. For each sample, 500 g of topsoil (0–10-cm depth) were collected. Nine soil attributes - available nitrogen, exchangeable potassium, extractable phosphorus, organic matter, soil pH, total potassium, total nitrogen, total phosphorus and soil bulk density - were analyzed as described by Liu *et al.* [41]. We used Kriging interpolation, to generate maps of the nine soil attributes using 20×20 m cells. To avoid over-fitting, we computed the principal components from the nine soil attributes and used the first three components as soil variables for the Bubeng plot. These condensed variables explained 83.5% of the total variance in the nine soil attributes. For the BCI plot, the Kriged estimates of Zn, Al, B, Ca, Fe, K, Cu, Mg, Mn, N, P, N (mineralisation) and pH values were calculated for 20×20 m cells. Again, we computed the first three principal components of the soil attributes, which explained 78.5% of the total variation, and used these to represent soil variables at the BCI plot.

### Testing size-class effect on the SARs: size-class categorization

To model size-class effect on species’ distributions, we classified trees into different DBH size-classes as an effective option for handling the tree size issue. We modelled species’ distributions using 3 DBH size-classes:, *viz.*

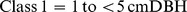


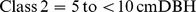


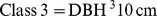



The three DBH class categorization was partly to be consistent with the studies by Hu et al. [33], [42], making our results comparable and ensuring that enough individuals can be found in the large DBH class. For each of the 3 DBH classes, species with an abundance of at least 50 were included in the point process model. When input that did not incorporate size effects was required these three size-classes were simply combined and conventionally designated, together,as Class ‘0’.

### Point process models

Following Shen *et al.* [21], we used the inhomogeneous Poisson process (to model environmental impacts), the homogeneous Thomas process (modelling dispersal) and the inhomogeneous Thomas process (modelling the joint-effects of environment and dispersal) to simulate tree species’ distributions. The simulations using the three point-process models were realized in the R statistical language (version 3.0.2) [43] using the R code of Shen et al. [21].

### Inhomogeneous poisson process

Models incorporating the inhomogeneous Poisson process use quadrat-based environmental variables to determine the density of target tree species in the corresponding quadrats. This model, accordingly, is useful for simulating species/habitat associations and we have used this model to determine the contribution of niche differentiation to species assembly. It employs the following function:
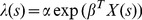
(1)where 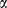
>0, 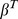 is a vector of regression parameters, 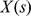 is a vector from the matrix of environmental variables - topographic and soil variables in this study.

### Thomas process models

Both homogeneous and inhomogeneous Thomas processes are varieties of Cox processes that drive patterns of clumping [21]. They can be used, therefore, to simulate processes generating patterns of aggregation [28], [44]. We used these models to simulate how parent trees dispersed offspring around themselves. We used the homogeneous version to explain dispersal-only limitation and the inhomogenous version to simulate the joint-effect of dispersal and environment. These models simulate the contribution to species assembly of neutral process and the joint-effect of niche and neutral process, respectively [21]. The Thomas point process *X* was a superposition of ‘mother’ points *c* in a stationary Poisson point process of intensity 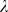, with associated ‘offspring’ clumps *Xc viz.:*

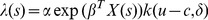
(2)where 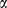 >0, 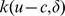 is a probability density function depending on a parameter *δ* >0 determining the spread of offspring points around *c*. 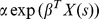 represents the covariance between event density and environment at point *s*. When 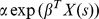 = 1, the function represents an *homogeneous* Thomas process; when the value is greater than unity, then the function is an *inhomogeneous* Thomas process. A more detailed description of the algorithms, parameter estimation and R-code for these three models can be found in Shen *et al.* [21].

### Simulating entire communities

To produce simulated communities incorporating the effects of size-classes, we firstly generated 100 simulated communities using a point process model for each of the three DBH classes: then by randomly superimposing the simulated assemblages of classes 1 to 3 together, 100 simulated communities with the size-class effect were created for each point process model. This does not model species interactions and intraspecific relationships among different size-classes. To represent simulated communities without the size-class effect, 100 simulated communities were generated directly using point process models of Class 0 (that is: with the three size-classes combined).

An SAR was constructed by generating an increasing series of 200 different sizes of randomly selected rectangular sampling cells from each specific simulated or real community [21]. The performance contrasts for these SARs between the point process models with and without the size-class effect were compared using an approximation of Akaike’s information criterion (*AIC*) [45]. As there were 100 simulated communities for each point process model, a Kruskal-Wallis rank sum test was used to identify the statistical difference between the *AIC* values of point process models with and without size-class effect. We also used *AIC* values to compare the performance of the three models. The *AIC* values of the three models can be approximated from the following formula [23], [45]:
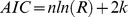
(3)where *n* is the number of sampled areas and equals 200, *R* is the sum of residual squares, and *k* is the number of parameters. The number of parameters of the inhomogeneous Poisson process, the homogeneous Thomas process and the inhomogeneous Thomas process are 2, 3 and 5, respectively [21]. As we had 100 simulated communities for each of the three point process models for each DBH class, we computed SAR *AIC* values for each simulated community. Again, we a Kruskal-Wallis rank sum test to compare the SAR *AIC* value differences between the point process models with and without the effect of size-class. We used pairwise Wilcoxon rank sum tests to assess the differences in SAR *AIC* values among the three point-process models for each DBH class.

### Testing size-class effect on the g-function

To evaluate the effect of incorporating size-classes on the point patterns of species’ distributions, the pair correlation function (i.e., the g-function) was computed for all the simulated and real species’ distributions. The g-function may effectively identify the occurrence of point-point interactions at a certain scale and evaluate the degree of aggregation. The g-function statistic is defined as
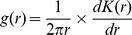
(4)where *g(r)* is the g-function, *K(r)* is Ripley’s K-function, and *r* is distance [46].

Specifically, we first computed the g-function value for each species of all the simulated and real communities at circumferences with a series of radii at 5, 10, 15, 20, 25, 30, 35, 40 and 45 m. To compare the degree of conspecific aggregation of the simulated distributions with and without the effect of size-class at the community level, we fitted them to the observed g-function using major axis regression [47]. Using this technique, if the fitted line is below the identity line (that is: where the fitted value is equal to the observed value of the g-function), then the point process model underestimates the degree of spatial aggregation: alternatively, the model overestimates the degree of spatial aggregation. By displaying the fitted lines of all the point process models against the identity line, we can identify the degree of deviation of the simulated patterns from the real data. In a manner similar to that used to calculate the SAR *AIC* values (see above), we computed the g-function *AIC* values to determine the performance of the three models. As before, we used Kruskal-Wallis rank sum tests to compare the differences in the *AIC* values of *g(r)* with and without size-class effects. We used pairwise Wilcoxon rank sum tests to evaluate differences in the *AIC* values of *g(r)* among the three point process models for each DBH class. The calculation of the *g(r)* and major axis regressions were also implemented in the R statistical language using the *spastat* [48] and *lmodel2* [49] packages, respectively.

## Results

### The SAR results

**Figure 1:**
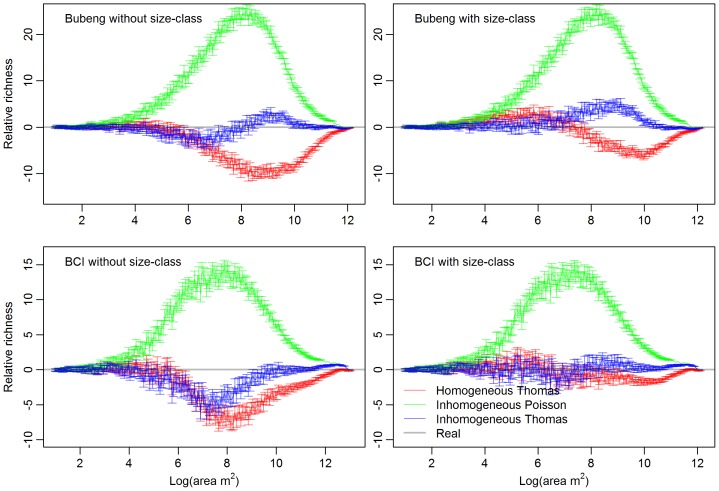
The observed and predicted species–area curves for the three process models with and without the size-class effect for Class 0 (that is: size-classes 1, 2 and 3 combined) of the Bubeng plot and the BCI plot. The bars represent 95% confidence intervals.

**Figure 2:**
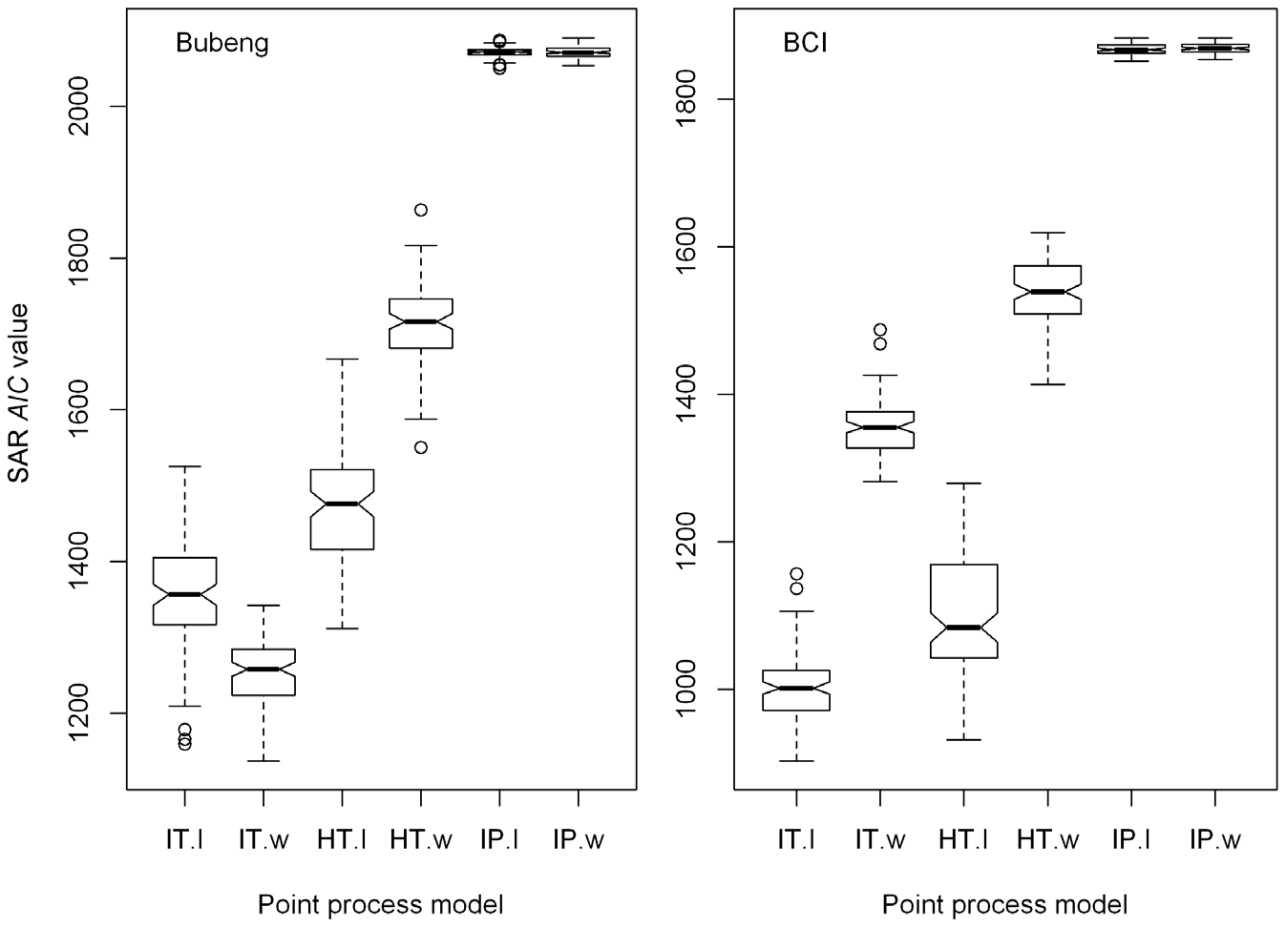
Boxplots of *AIC* value distributions of the SARs generated by the three process models with and without the size-class effect at Class 0 (that is: size-classes 1, 2 and 3 combined) forthe Bubeng plot and the BCI plot. HT.l = the homogeneous Thomas process with size-class effect; HT.w = the homogeneous Thomas process without size-class effect; IP.l = the inhomogeneous Poisson process with size-class effect; IP.w = the inhomogeneous Poisson process without size-class effect; IT.l = the inhomogeneous Thomas process with size-class effect; and IT.w = the inhomogeneous Thomas process without size-class effect.

For the Bubeng plot, 158 tree species were used in the analyses designated as size-class 0. Out of these species, there were 148 species in size-class 1, 43 in class 2 and 52 in class 3. For the BCI plot, there were 169 tree species at class 0, 161 tree species at class 1, 83 tree species at class 2, and 77 tree species in class 3. Figure 1 shows the SAR patterns of the simulated communities produced using the three point-process models with and without an size-class effect for Class 0 of the Bubeng plot and the BCI plot. Using the inhomogeneous Poisson process, there was almost no shape difference in shape in the relative SAR patterns between the simulated communities with and without an size-class effect for both plots. For the inhomogeneous Thomas and the homogeneous Thomas processes, there were significant shape differences in the relative SAR patterns between the simulated communities with and without size-class effect across the two plots. The SAR *AIC* value distributions at class 0 and the Kruskal-Wallis rank sum test results on them verified the differences statistically (Fig. 2 and Table 1). The p-values of Table 1 suggest that the SAR *AIC* values are remarkably reduced when size-class effects are introduced into the homogeneous Thomas process for class 0 of the Bubeng plot and the BCI plot. However, the SAR *AIC* values were only similarly reduced by the size-class effect for the inhomogeneous Thomas process for class 0 for the BCI plot.

**Table 1:** The p-values from Kruskal-Wallis rank sum tests on SAR and *g(r) AIC* values across the point process models with and without size-class effect at DBH class 0 (that is: size-classes 1, 2 and 3 combined).

Summary statistics	IP	HT	IT
SAR *AIC*	0.828	<<0.001	<<0.001
	0.290	<<0.001	<<0.001
*g(r) AIC*	0.769	0.807	0.843
	0.474	0.680	0.092

Note: IP = the inhomogeneous Poisson process, HT = the homogeneous Thomas processt, IT = the inhomogeneous Thomas process.

**Figure 3:**
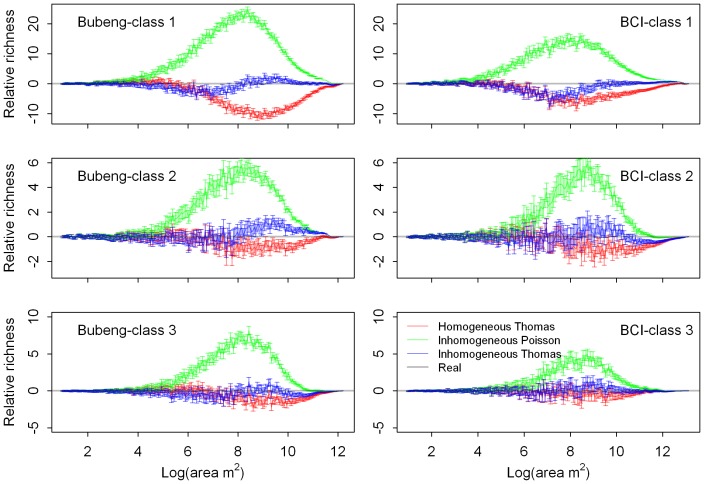
The observed and predicted species–area curves for the three process models for size-classes 1, 2 and 3 of the Bubeng plot and the BCI plot. The bars represent 95% confidence intervals.

**Figure 4:**
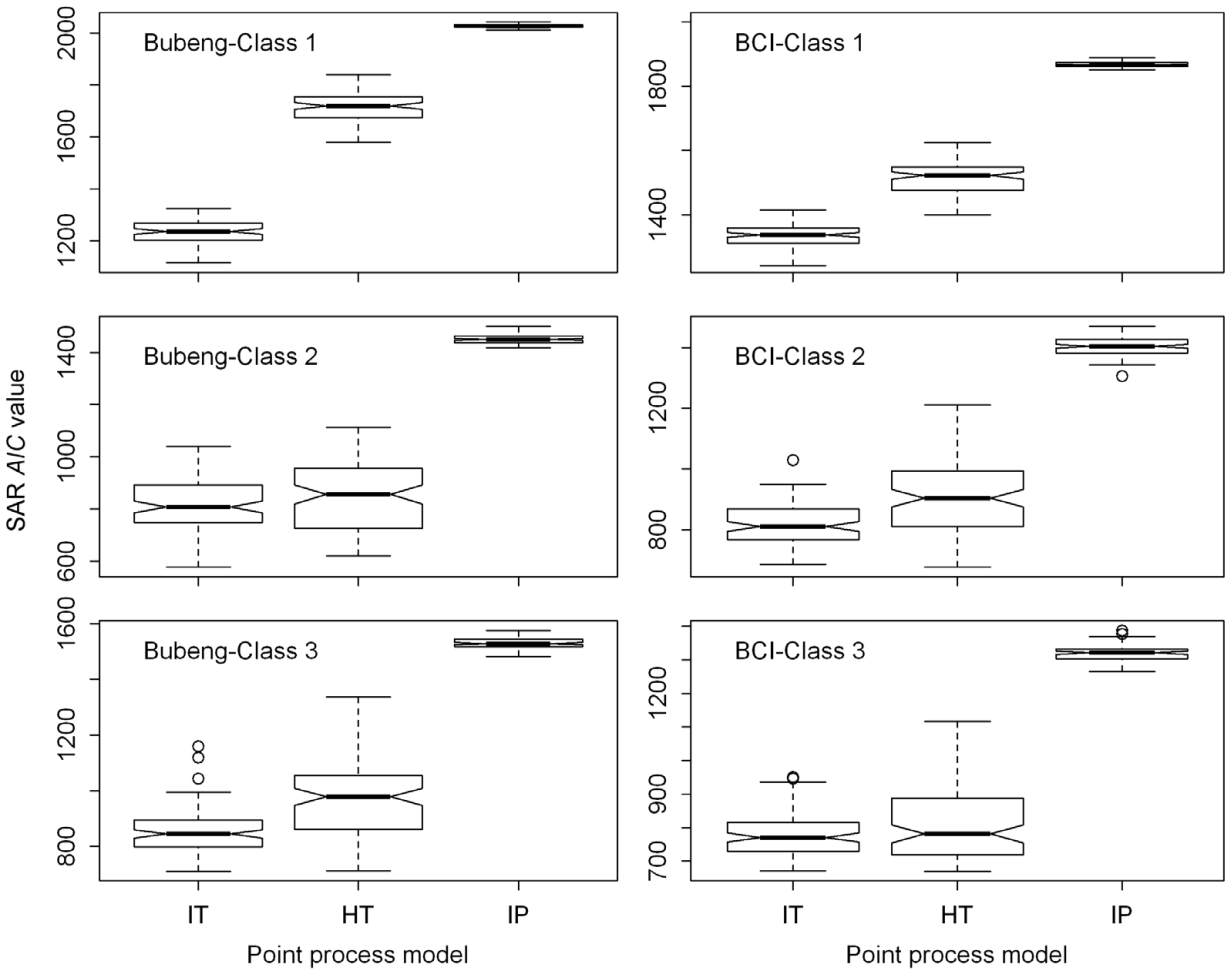
Boxplots of *AIC* value distributions of the SARs generated by the three process models for size-classes 1, 2 and 3 of the Bubeng plot and the BCI plot. HT = the homogeneous Thomas process; IP = the inhomogeneous Poisson process; IT = the inhomogeneous Thomas process.

The inhomogeneous Poisson process always overestimated the SAR patterns from class 0 to 3 for both plots (Figs. 1 and 3). Among the three point-process models, the inhomogeneous Thomas process always led to the most accurate SAR estimation from Class 0 to 3 (Figs. 1–4, Tables 2 and Table S1). As the DBH class increased, so the SAR *AIC* value differences between the homogeneous Thomas and the inhomogeneous Thomas processes were gradually reduced, again for both plots (Fig. 4 and Table 2). Because the inhomogeneous Thomas process modelled the joint-effects of environment and dispersal, the reason for this trend may be that dispersal is always more influential than environment.

**Table 2:** The p-values of pairwise Wilcoxon rank sum tests on SAR and *g(r) AIC* values among the three point process models for the three DBH classes.

			Class 1		Class 2		Class 3	
Summary statistics	Site	Model	IT	HT	IT	HT	IT	HT
SAR	Bubeng	HT	<<0.001	–	0.144	–	<<0.001	–
		IP	<<0.001	<<0.001	<<0.001	<<0.001	<<0.001	<<0.001
	BCI	HT	<<0.001	–	<<0.001	–	<<0.001	–
		IP	<<0.001	<<0.001	<<0.001	<<0.001	<<0.001	<<0.001
*g(r)*	Bubeng	HT	<<0.001	–	<<0.001	–	<<0.001	–
		IP	<<0.001	0.976	<<0.001	0.001	<<0.001	0.209
	BCI	HT	<<0.001	–	<<0.001	–	<<0.001	–
		IP	<<0.001	0.001	<<0.001	<<0.001	<<0.001	<<0.001

Note: See Table 1 for the abbreviations.

### The *g(r)* results

**Figure 5:**
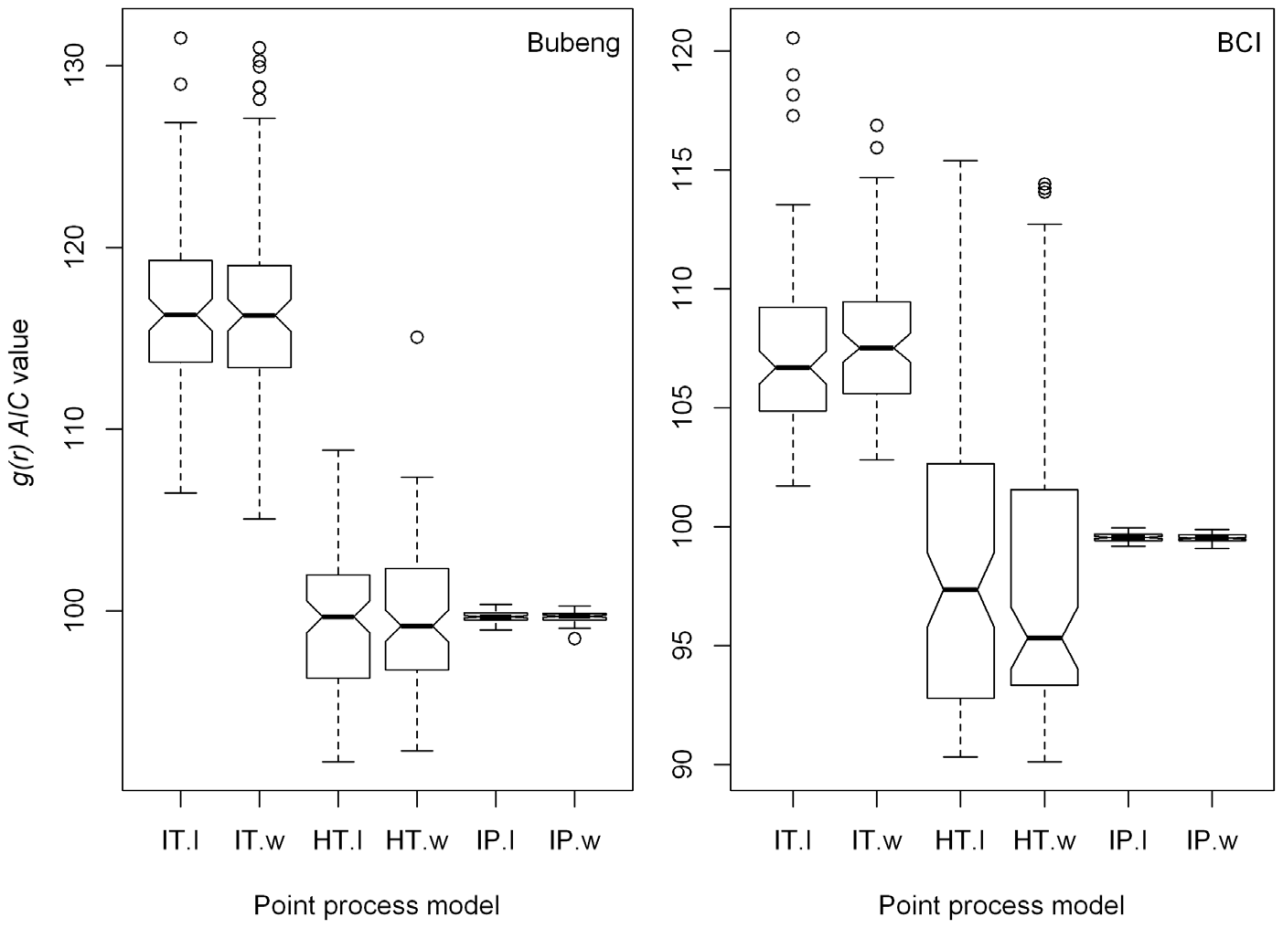
Boxplots of *g(r) AIC* value distributions generated by the three processe models with and without the size-class effect at Class 0 (that is: size-classes 1, 2 and 3 combined) of the Bubeng plot and the BCI plot.

**Figure 6:**
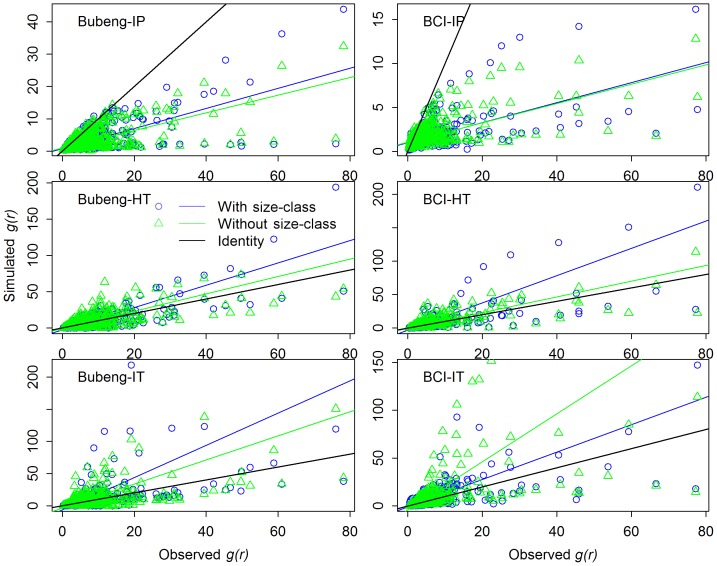
Comparison of observed *g(r)* of species at DBH Class 0 (that is: size-classes 1, 2 and 3 combined) with *g(r)* simulated by three point process models with and without size-class effect. Abbreviations as for Figure 4.

There was no significant difference in *g(r) AIC* values between the simulated species’ distributions with and without size-class effects for any of the three point-process models for Class 0 of the two plots (Fig. 5 and Table 1). In contrast to the SAR results, the *g(r) AIC* values of from the model based on the inhomogeneous Thomas process were always the highest of any of the three models evaluated and the difference is significant for Class 0 (Fig. 5 and Table S1). This was also true for Classes 1 to 3 (Fig. S2 and Table 2). The major axis regression results on degree of conspecific aggregation showed that the point-process models with size-class effects overestimated the degree of conspecific aggregation compared with those without the size-class effect (Fig. 6).

Of the three point-process models, the lines fitted using major axis regression from the inhomogeneous Poisson process were below the identity line for Classes 0 to 3 (Figs. 6 and S1). This suggests that the simulated patterns of species’ distributions using this model were always less clumped than the real species’ distributions. Conversely, the other two point-process models always overestimated the degree of conspecific aggregation across all size-classes (Figs. 6 and S1).

## Discussion

### Performances of the models

The SAR and, especially, the g-function results (Figs. 2, 4, 5, S1 and S2), suggest that the model based on the homogeneous Thomas process performed well across all DBH classes for the two forests. Although the SAR *AIC* values using the homogeneous Thomas process were not the lowest of the three models, the differences in *AIC* values between the homogeneous Thomas and the inhomogeneous Thomas process gradually decrease (Figs. 3 & 4). On the other hand, the ecological factors inherent in the models based on inhomogeneous Thomas processes and the inhomogeneous Poisson process play important roles in determining spatial patterns at the community and species levels, respectively. In previous studies models based on the inhomogeneous Thomas process are close to the best performing of the three basic kinds of point process models that have investigated SARs and distance decay curves at the community level [20], [21], with L-function patterns at species level [25], with species abundance distributions, and with nearest-neighbor distance functions at both the species and community levels [23]. This is not the case, however, in our study.

Unlike other studies only that of Wang *et al.* [20], for the Changbaishan 25 ha plot in China, have found that the inhomogeneous Poisson process generated the best distance-decay curve for larger trees (DBH ≥10 cm). With respect to the *g(r)* results (Figs. 6 & S1), for instance, for the Bubeng plot, the point patterns of simulated distributions of *Mezzettiopsis creaghii*, *Alchornea tiliifolia*, *Castanopsis echidnocarpa* and *Knema furfuracea* produced by the inhomogeneous Thomas process are much more aggregated than their real distributions (Figs. S3–S6). In fact, of the three models, the inhomogeneous Poisson process can even produce the best *g(r)* patterns at the species level (Figs 5 & S2).

A comparison of the three point-process models within our study indicates that the summary characteristics used can strongly affect the study results and the conclusions. This is because critical characteristics of the complex patterns underlying species’ distributions may be emphasized differently by different summary characteristics [24]. Our conclusion, based on SARs and *g(r)* functions which detect spatial patterns at community and species levels, may be more robust than those based on summary characteristics that incorporate species’ distribution patterns at either, but not both, of community or species levels.

### Contributions of the ecological processes

After synthesizing the results of the SARs and *g(r)*, reflecting community and species levels patterns, respectively, we have demonstrated that dispersal largely controls species’ distributions within the two forests evaluated. Moreover, as it shown in Figures 3 & 4, the role of dispersal in driving species’ distributions increases with size-class increasing, whereas that of environment decreases. This pattern is consistent with a recent finding that the contributions of neutral processes to tree species survival shift with the change from juvenile to reproductive stages at the BCI plot [10]. At the Bubeng plot, it has once been verified, using regression and ordination methods on the tree lattice data, that dispersal is the dominant process in shaping species’ distributions [33], [42]. At the BCI plot, Levine and Murrell [50] have suggested that dispersal is important for species’ distributions at the species level. Our results demonstrate that dispersal is also crucial in determining species’ distributions at both community and species levels. Moreover, the finding that dispersal dominates the structuring of species’ distributions of trees from small to large size is consistent with the reports by Seidler and Plotkin [1] and Leithead et al. [51]. Specifically, Seidler and Plotkin [1] demonstrated that species’ distributions are related to dispersal mode from saplings to mature trees and Leithead et al. [51] showed that the dispersal process is important for species spatial patterns for the BCI plot. Our results also suggest that dispersal can be the principal process shaping community spatial pattern on plots with large elevation ranges (160 m for Bubeng). This is also true for the Gutian 24 ha plot which has an elevational range of 268.6 m [52]. In contrast, at the Sinharaja 25 ha plot with only a 151 m elevation range, Gunatilleke et al. [53] demostrate that environment dominantes species distributions. The contrasting results from these plots indicate that the contribution of environment to species distribution does not necessarily relate to elevation range. To sum up, dispersal is a dominant process in shaping species’ distributions in both forests.

### Size-class effects

The effects of size-class serve as an essential supplementary mechanism for understanding the patterns of species area relationships. Our results show clearly that the incorporation of size-classes improves the accuracy of SAR estimation using models based on homogeneous Thomas processes for the two forests, and the accuracy of those using inhomogeneous Thomas process at the BCI plot (Fig. 2). As we have already indicated, dispersal is a dominant process determining species’ distributions; we hypothesize, therefore, that several crucial mass dispersal phases at different life stages that shape the real species’ distributions in the two forests may exist. For the BCI plot, the finding by Seidler and Plotkin [1] that tree distributions from saplings to adults are strongly related to dispersal provides direct evidence for this hypothesis. Previous studies also demonstrated that, at the Bubeng plot, the distributions of tree species are predominantly determined by neutral spatial processes across size-classes [42]. In summary, we suggest that the size-class effect is important for determining tree species’ distributions through influencing dispersal characteristics from juvenile to adult trees.

On the other hand, the concordant SAR patterns produced using the inhomogeneous Poisson process with and without size-class effects suggest that species’ habitat preferences at different DBH classes are almost uniform. This is consistent with the finding that most of the species tend to maintain their habitat preferences across different DBH size-classes in a 24-ha subtropical forest plot in China [6]. Why do the habitat preferences of species become homogenous across different size-classes? By studying habitat-driven assemblages of species in three size-classes from the BCI plot, Kanagaraj *et al.* [10] found that species’ habitat preferences become weaker as they age. Figures 3 and 4 further suggest that such habitat preferences are basically formed at an early stage of life. This is because SAR patterns of the inhomogenous and homogenous Thomas processes only differ greatly from each other at Class 1, and little for Classes 2 and 3. This is consistent with the predictions of the theory of regeneration niche differentiation which predicts that species’ habitat preferences most likely form at an early stage of life and this preference is maintained through later size stages [54]. What we have shown is consistent with the theory when a rigorous quantitative statistical analysis is applied.

The size-class effect might be affected by the location of the plot (i.e. via its environmental heterogeneity). As it shown in Figure 2, the influence of size-class shows, on the one hand, that the trend of impact on dispersal processes is the same but its influence on the joint process (environment+dispersal) exhibits differential impacts on the two plots. This suggests that the addition of environmental factors to the point process model may result in a different performance of the joint process. As the site location of interest is the major source of environmental variation, using more plots and conducting meta-analyses may shed light on to this issue.

The importance of the size-class effect on species’ distributions at the community level is significantly different from that at the species level, having a significant impact on the SAR patterns but not on the *g(r)* patterns in either of the two forests examined. A possible reason for this distinction is that SARs and *g(r)* functions reflect species’ distributions at different scales. SARs are related to the spherical contact distribution, which fundamentally describes the vacant area between clumps [28] and thus is less capable of reflecting the pattern at smaller species-level scales. On the contrary, the *g(r)* function describes the details of clumps of individual species and focuses on patterns at that scale [31]. It is most likely, therefore, that the effect of size-class principally influences species’ distributions at relatively large scales.

## Conclusion

The mechanisms underlying the community assembly of tree species remain controversial: niche-based mechanisms [17], neutral mechanisms [12], and joint mechanisms incorporating niche and neutral processes [52] remain under discussion. Previous studies using point-process models to investigate community structure report that the joint-effect of environment and dispersal are dominant in generating community characteristics across tropical, subtropical and temperate forests [20], [21], [23], [25]. Our results, however, indicate that dispersal is the more dominant process in the determination of the spatial patterns of species across size-classes at both species and community levels for both the rainforests examined. We argue that evaluating the relative importance of environment and/or dispersal using only summary characteristics at the species or community level may lead to bias. Many studies report that the mechanisms regulating species’ distributions vary across size-classes [5], [6], [10], but we find that dispersal is always the predominant process that regulates the distributions of tree species from juvenile to adult stages. Habitat preferences of species usually develop at an early stage of life and weaken thereafter, as DBH increases. We conclude that it is critical to use summary characteristics at both community and species level to identify the ubiquitous mechanisms that determine species’ distributions. Size-class effect contribute to species’ assembly through differential dispersal which, we contend, is the most important process in both forests studied.

## Supporting Information

Figure S1 Comparison of observed *g(r)* of species at DBH class 1 to 3 with simulated *g(r)* by the three point process models.
(TIF)

Figure S2 Boxplots of *g(r) AIC* value distributions generated by the three process models from class 1 to 3 of the Bubeng plot and the BCI plot. See Figure 4 for the abbreviations.
(EPS)

Figure S3 The real distribution of *Mezzettiopsis creaghii* in the Bubeng plot; its distributions predicted from the inhomogeneous Poisson process, the inhomogeneous Thomas process and the homogeneous Thomas process with the size-class effect; and, its distributions predicted from the inhomogeneous Thomas process and the homogeneous Thomas process scenarios without the size-class effect. The units of x and y axes are meter. See Figure 4 for the abbreviations.
(TIF)

Figure S4 The real distribution of *Alchornea tiliifolia* in the Bubeng plot; its distributions predicted from the inhomogeneous Poisson process, the inhomogeneous Thomas process and the homogeneous Thomas process with the size-class effect; and its distributions predicted from the inhomogeneous Thomas process and the homogeneous Thomas process scenarios without the size-class effect. The units of x and y axes are meter. See Figure 4 for the abbreviations.
(TIF)

Figure S5 The real distribution of *Castanopsis echidnocarpa* in the Bubeng plot; its distributions predicted from the inhomogeneous Poisson process, the inhomogeneous Thomas process and the homogeneous Thomas process with the size-class effect; and its distributions predicted from the inhomogeneous Thomas process and the homogeneous Thomas process scenarios without the size-class effect. The units of x and y axes are meter. See Figure 4 for the abbreviations.
(TIF)

Figure S6 The real distribution of *Knema furfuracea* in the Bubeng plot; its distributions predicted from the inhomogeneous Poisson process, the inhomogeneous Thomas process and the homogeneous Thomas process with the size-class effect; and its distributions predicted from the inhomogeneous Thomas process and the homogeneous Thomas process scenarios without the size-class effect. The units of x and y axes are meter. See Figure 4 for the abbreviations.
(TIF)

Table S1 The p-values of pairwise Wilcoxon rank sum tests on SAR and *g(r) AIC* values among the three point process models at DBH class 0 (that is: size-classes 1, 2 and 3 combined).
(DOC)

Data S1 Dataset for the Bubeng 20 plot.
(XLSX)
