# Supplementary figures and images for: Size-Class Effect Contributes to Tree Species Assembly through Influencing Dispersal in Tropical Forests
Source: PLoS One. 2014 Sep 24;9(9):e108450. doi: 10.1371/journal.pone.0108450 (PMC4177404; doi:10.1371/journal.pone.0108450)

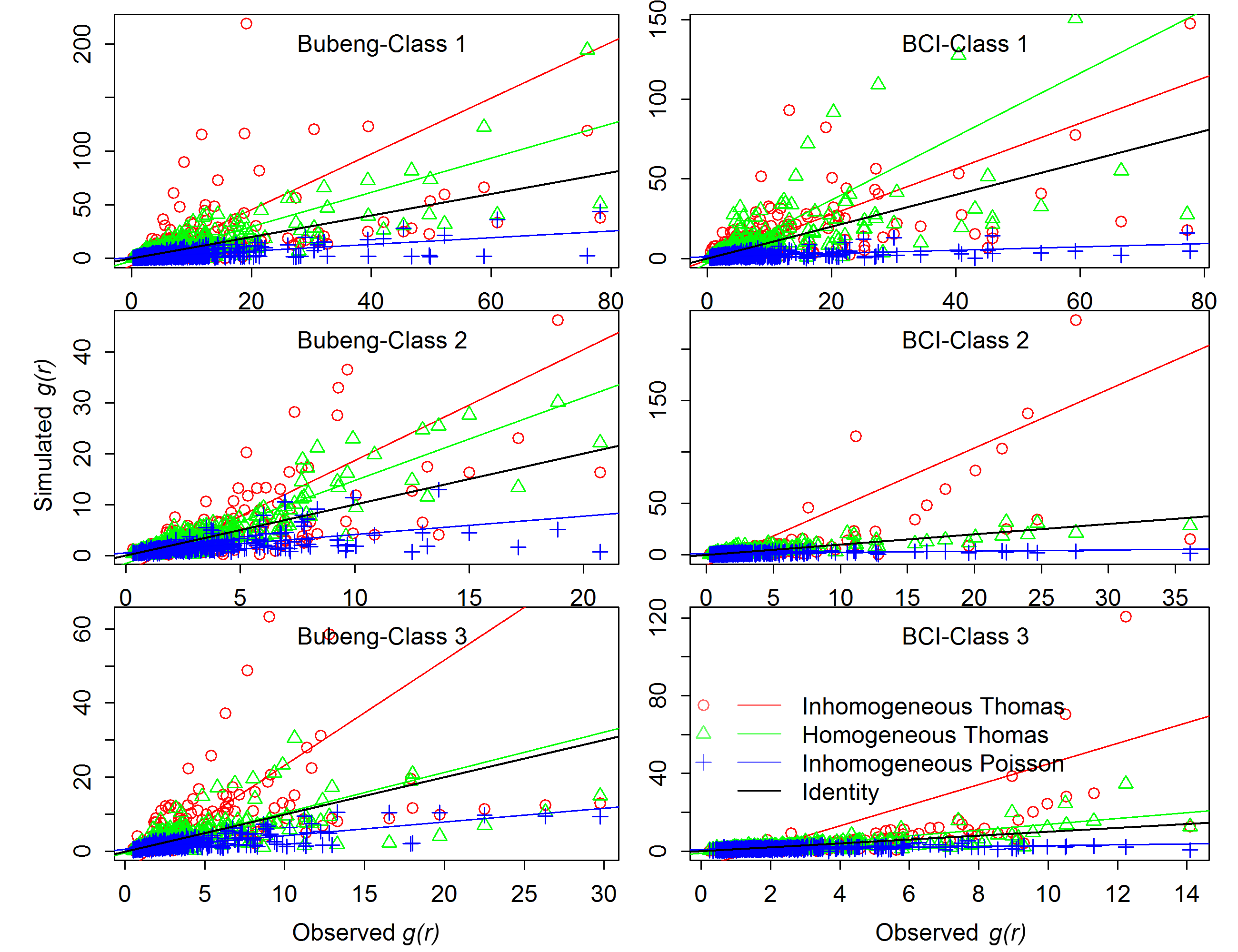

Supplement: Figure S1 — Comparison of observed g(r) of species at DBH class 1 to 3 with simulated g(r) by the three point process models. (TIF) [file pone.0108450.s001.tif]

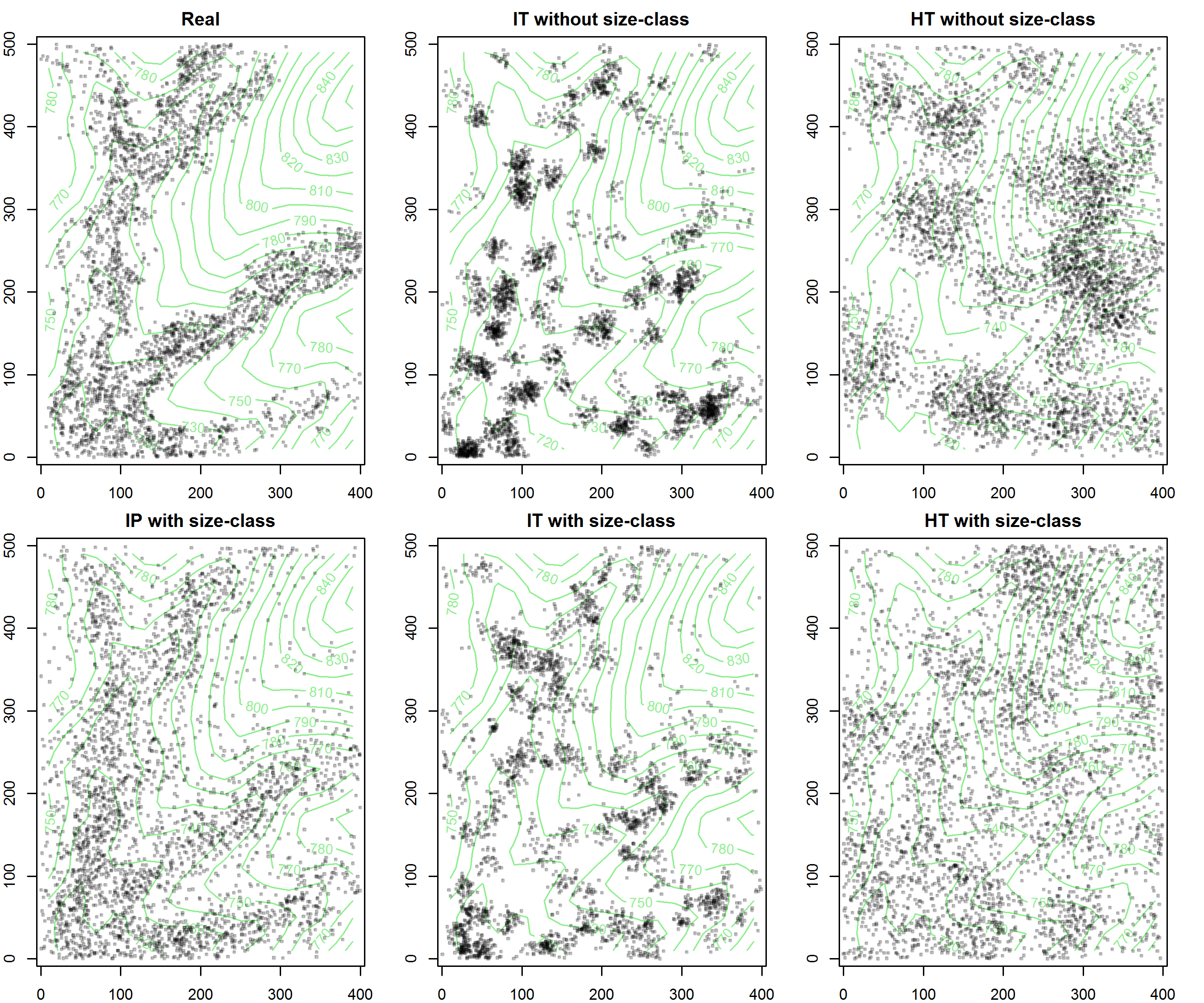

Supplement: Figure S3 — The real distribution of Mezzettiopsis creaghii in the Bubeng plot; its distributions predicted from the inhomogeneous Poisson process, the inhomogeneous Thomas process and the homogeneous Thomas process with the size-class effect; and, its distributions predicted from the inhomogeneous Thomas process and the homogeneous Thomas process scenarios without the size-class effect. The units of x and y axes are meter. See Figure 4 for the abbreviations. (TIF) [file pone.0108450.s003.tif]

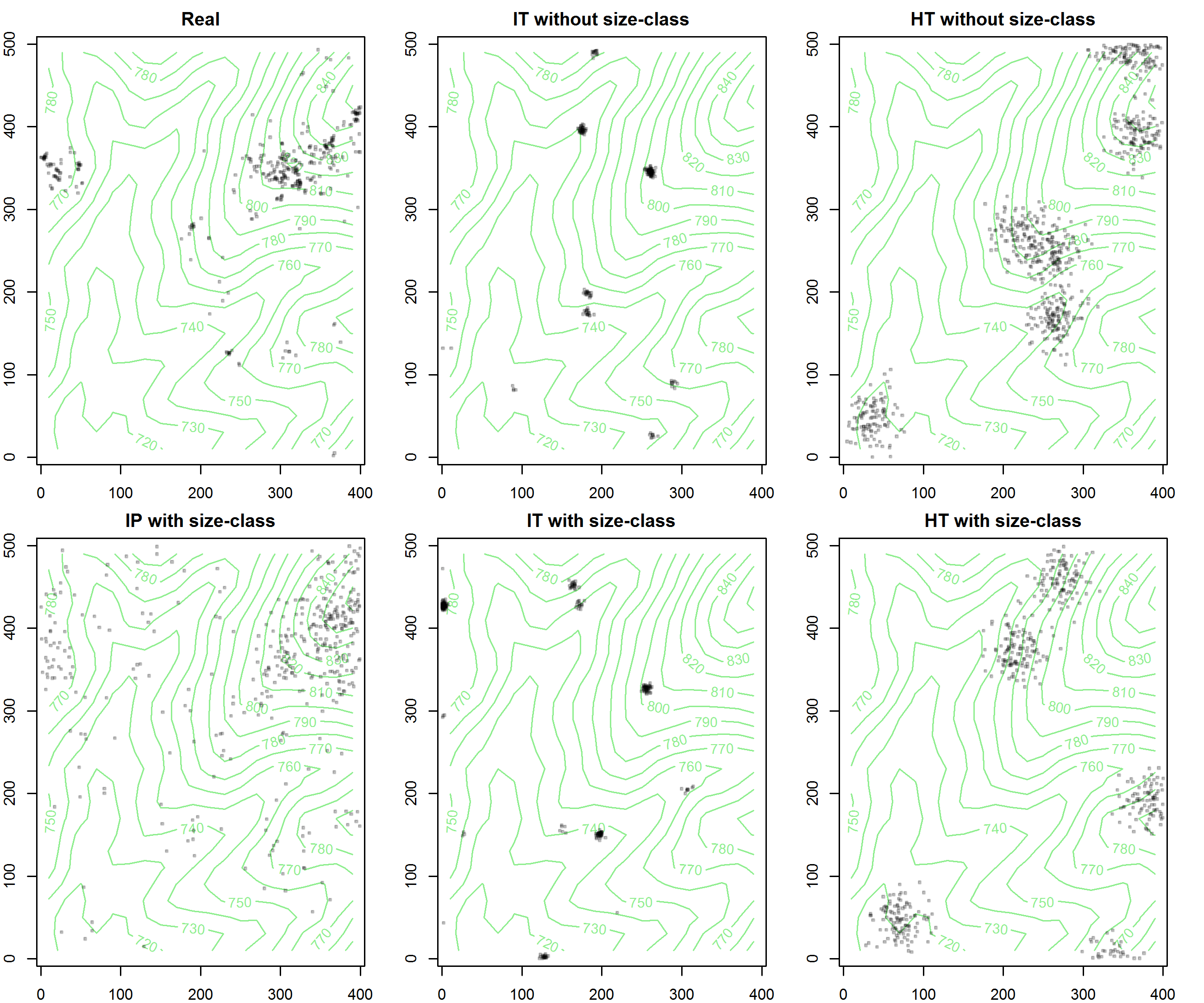

Supplement: Figure S4 — The real distribution of Alchornea tiliifolia in the Bubeng plot; its distributions predicted from the inhomogeneous Poisson process, the inhomogeneous Thomas process and the homogeneous Thomas process with the size-class effect; and its distributions predicted from the inhomogeneous Thomas process and the homogeneous Thomas process scenarios without the size-class effect. The units of x and y axes are meter. See Figure 4 for the abbreviations. (TIF) [file pone.0108450.s004.tif]

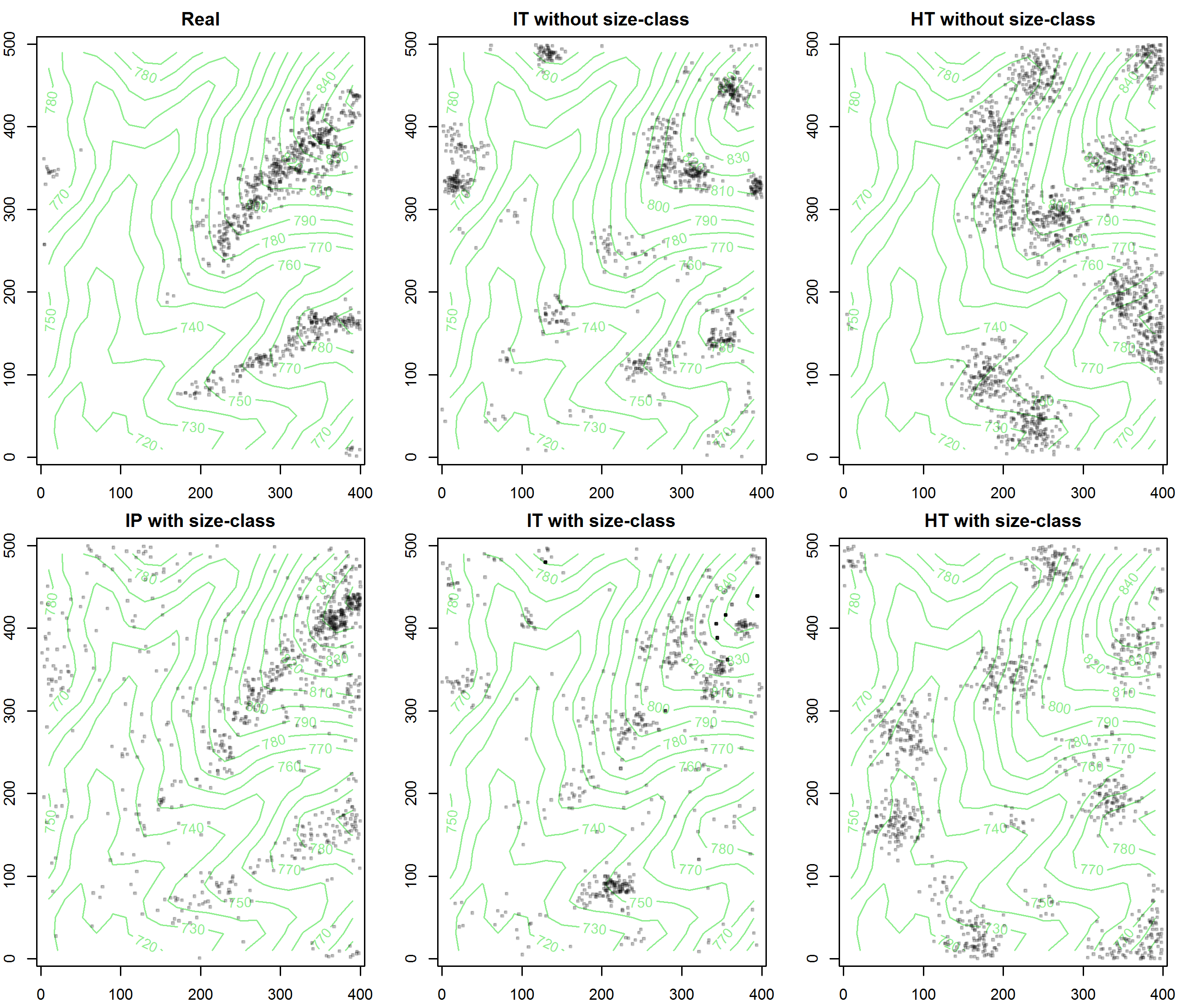

Supplement: Figure S5 — The real distribution of Castanopsis echidnocarpa in the Bubeng plot; its distributions predicted from the inhomogeneous Poisson process, the inhomogeneous Thomas process and the homogeneous Thomas process with the size-class effect; and its distributions predicted from the inhomogeneous Thomas process and the homogeneous Thomas process scenarios without the size-class effect. The units of x and y axes are meter. See Figure 4 for the abbreviations. (TIF) [file pone.0108450.s005.tif]

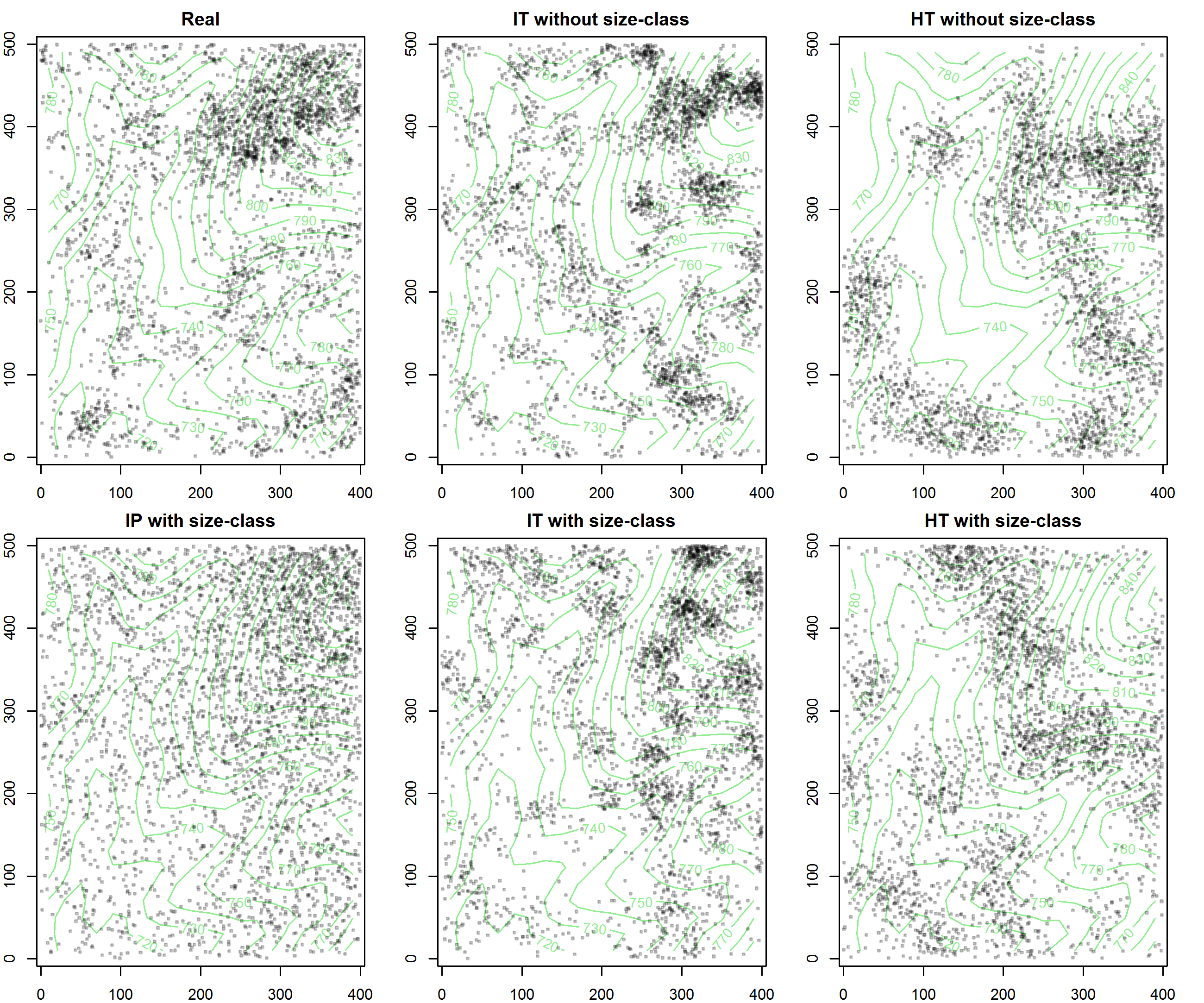

Supplement: Figure S6 — The real distribution of Knema furfuracea in the Bubeng plot; its distributions predicted from the inhomogeneous Poisson process, the inhomogeneous Thomas process and the homogeneous Thomas process with the size-class effect; and its distributions predicted from the inhomogeneous Thomas process and the homogeneous Thomas process scenarios without the size-class effect. The units of x and y axes are meter. See Figure 4 for the abbreviations. (TIF) [file pone.0108450.s006.tif]
