# Supplementary material for: Size-Class Effect Contributes to Tree Species Assembly through Influencing Dispersal in Tropical Forests
Source: PLoS One. 2014 Sep 24;9(9):e108450. doi: 10.1371/journal.pone.0108450 (PMC4177404; doi:10.1371/journal.pone.0108450)
Supplement: Table S1 — The p-values of pairwise Wilcoxon rank sum tests on SAR and g(r) AIC values among the three point process models at DBH class 0 (that is: size-classes 1, 2 and 3 combined). (DOC) [file pone.0108450.s007.doc]

Table S1 The p-values of pairwise Wilcoxon rank sum tests on SAR and *g(r) AIC* values among the three point process models at DBH class 0 (that is: size-classes 1, 2 and 3 combined).

| Summary statistics | Site | Model | IT.l | HT.l |  | IT.w | HT.w |
| --- | --- | --- | --- | --- | --- | --- | --- |
| SAR | Bubeng | HT.l | <<0.001 | - | HT.w | <<0.001 | - |
|  |  | IP.l | <<0.001 | <<0.001 | IP.w | <<0.001 | <<0.001 |
|  | BCI | HT.l | <<0.001 | - | HT.w | <<0.001 | - |
|  |  | IP.l | <<0.001 | <<0.001 | IP.w | <<0.001 | <<0.001 |
| *g(r)* | Bubeng | HT.l | <<0.001 | - | HT.w | <<0.001 | - |
|  |  | IP.l | <<0.001 | 1 | IP.w | <<0.001 | 1 |
|  | BCI | HT.l | <<0.001 | - | HT.w | <<0.001 | - |
|  |  | IP.l | <<0.001 | 1 | IP.w | <<0.001 | 1 |

Note: HT.l = the homogeneous Thomas process with size-class effect, HT.w = the homogeneous Thomas process without size-class effect, IP.l = the inhomogeneous Poisson process with size-class effect, IP.w = the inhomogeneous Poisson process without size-class effect, IT.l = the inhomogeneous Thomas process with size-class effect, and IT.w = the inhomogeneous Thomas process without size-class effect.
